# Introduction to molecular replacement: a time perspective

**DOI:** 10.1107/S2059798321004368

**Published:** 2021-06-18

**Authors:** Eleanor Dodson

**Affiliations:** a Department of Chemistry, University of York, Heslington, York YO10 5DD, United Kingdom

**Keywords:** crystallographic theory, molecular replacement, test cases, history, scoring functions, crystallographic equations, crystal phasing

## Abstract

This article provides an introduction to the crystal phasing technique known as molecular replacement. The available software is reviewed, and the prospects for future developments are considered. Several examples are described in detail to illustrate potential problems. A brief account of past progress is included. The basic crystallographic equations underlying the procedures are given in an appendix.

## Abstract 2

This article lists the basic crystallographic equations underlying molecular-replacement software. The prospects for future developments are considered, and a brief account of past progress is included.

## 1. Introduction   

The underlying reason for embarking on most structural biology studies is to add to one’s understanding of how this particular macromolecule contributes to the machinery of a living cell. X-ray crystallography can provide a three-dimensional image of the molecule to guide this understanding, using the observed diffraction and derived phases.

**Figure 1:**
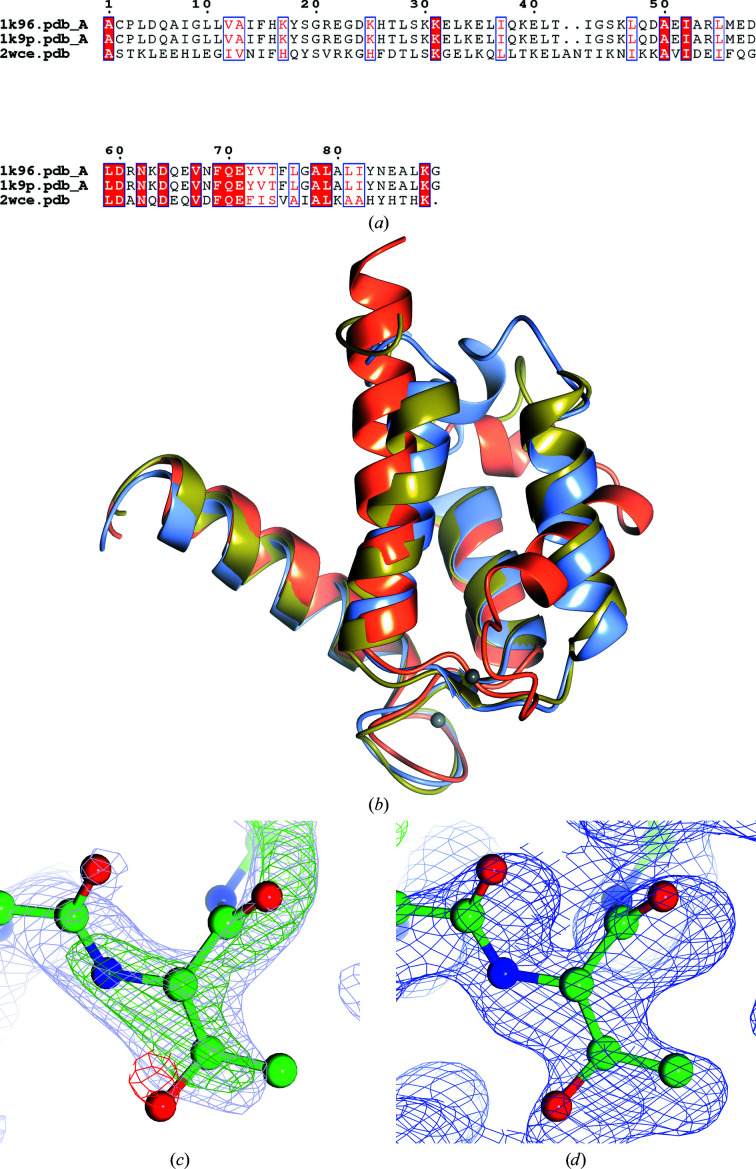
(*a*) Sequence alignment and (*b*) overlap of PDB entries 2wce (blue), 1k9p (yellow) and 1k96 (brown), showing the domain movement between PDB entries 2wce and 1k96. (*c*) The difference electron density for Thr43, missing from the search model, after initial refinement to *R* and *R*_free_ factors of 46% and 49%, respectively. (*d*) Final electron-density map for Thr43 after *Buccaneer* rebuild: *R* = 22%

This paper aims to briefly outline the basic crystallographic principles underlying the molecular-replacement (MR) technique, which is now the preferred method for obtaining initial phasing. The aim of the technique is to match a model with known structure to the X-ray observations measured from another crystal form containing a related molecule. If the known model can be rotated and translated as a rigid body to an approximately correct position in the new cell, then the phases generated from this imperfect model can kick-start the reconstruction of the molecule under investigation (Fig. 1 ▸). Details of the procedures have been described in various articles and reviews. Comprehensive coverage is given in the *Proceedings of the CCP4 Study Weekend* from 2008 (Evans & McCoy, 2008 ▸).

All crystallographic studies require consideration of the following four stages: I will discuss each under a separate heading.

(i) What is the **chemical composition** of the molecule that you hope to crystallize? Is it made up of amino acids only? Are there associated metals, ligands, nucleic acids and/or carbohydrates? Is there a known structure with similar components?
(ii) If the molecule can be crystallized and these crystals diffract, then what are the properties of your **diffraction images and the crystal lattice**?
(iii) Is it possible to position **a starting model** in the **crystal lattice**? This requires the use of molecular-replacement techniques to find plausible positions and a scoring system to rank likely solutions before proceeding to stage (iv).
(iv) Can you **bootstrap** from this preliminary model to an accurate final structure?

## 2. Crystallographic fundamentals   

Before discussing the techniques and scoring systems used for molecular replacement, it is useful to remind ourselves of the fundamental crystallographic equations. These are described in more detail in Appendix *A* [App appa] and touch on (i) the properties of a crystal, (ii) diffraction, (iii) the structure-factor equation, some effects of symmetry and origin shifts, (iv) electron-density maps and (v) Patterson maps.

### 2.1. Structure-factor equation   

For *N* atoms at positions **x**_*j*_ with form factor *f*_*j*_(*S*) and correction *T*_*j*_(*S*), a term that accounts for the falloff in scattering from thermal motion,

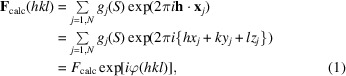

where *g*_*j*_(*S*) = *f*_*j*_(*S*)*T*_*j*_(*S*). *F*(*hkl*) and φ(*hkl*) are referred to as the structure-factor amplitude and phase, respectively.

The key point here is that each observed reflection will contain information about the position and temperature factor of every atom.

### 2.2. Electron-density equation   

The equation for the electron density is used to compute its value at **discrete regular divisions** (grid points) of the unit cell. If the phases are accurate, there will be a peak in the density when the **map coordinate** (*x*, *y*, *z*) is close to the **model coordinate** (*x*_*j*_, *y*_*j*_, *z*_*j*_):

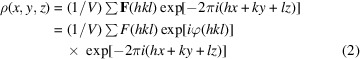

summed over all *h*, *k* and *l*.

### 2.3. Maximum-likelihood-weighted difference electron-density map   




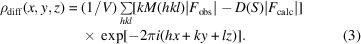

This map should show ONLY the differences between the true and observed models, with positive maxima where the atoms are ‘missing’ and negative minima if an atom in the model is in a wrong place (Robertson & Woodward, 1936 ▸).

Such maps are used to extend and correct coordinates (Fig. 1 ▸ *c*).

### 2.4. Patterson maps   




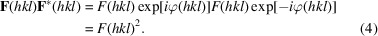

Calculating a map replacing **F**(*hkl*) with *F*(*hkl*)*F*(*hkl*)* and with **all phases zero** gives a map with peaks at all positions (**x**_*i*_ − **x**_*j*_), *i.e.* at the vector difference between any two atoms **x**_*j*_ and **x**_*i*_. Patterson interpretations can kick-start many phasing procedures (Patterson, 1934 ▸).

## 3. Investigating the known biochemical and structural information   

Most crystallographic projects are undertaken with some knowledge of the nature of the molecule(s) under investigation: typically, their sequence, any likely ligand and hence their molecular weight.

There are a wealth of freely available databases which can match sequences, either to all other published sequences or just to the sequences of known structures [for example, *HHpred* (Söding *et al.*, 2005 ▸; Remmert *et al.*, 2012 ▸) and *PHMMER* (Eddy, 2011 ▸)]. One or more of the set of structures with related sequences may well provide a suitable model for molecular replacement. Whether or not the model will lead to a molecular-replacement solution depends on the r.m.s. deviation of the model to the target, the fraction of the scattering that it represents and, importantly, the resolution of the data.

It is sensible to study the nature of the chosen model(s) and to carry out some bio-informatic analyses even before any crystal has grown.

Things to consider include the following.

(i) Has the structure already been solved and deposited? It can happen! (Simpkin *et al.*, 2020 ▸).
(ii) Is there a reasonably complete model with sequence identity better than 30%? For such a case, molecular replace­ment will probably be straightforward and the challenge will be to rebuild the new structure satisfactorily. This is always simpler with higher resolution observed data, so it is sensible to optimize the crystal and data quality.
(iii) After overlapping possible models it may be obvious that the models have matching domains but that these domains are arranged differently. Fig. 1 ▸(*b*) shows an example of this. The domains of S100 are very differently packed in the presence or absence of calcium. Using *MrBUMP* to select models, and *CCP*4*MG* to align and display them, provides a useful tool for analysing this (Keegan *et al.*, 2018 ▸). A tutorial is available at https://www.ccp4.ac.uk/schools/APS-2010/tutorials/mrbump/APS-MrBUMP-tutorial-2010.pdf.
(iv) Is the chosen model part of an oligomer: maybe a dimer, a trimer, a tetramer or even part of a circular complex such as GroEL, a dual-ringed structure with both rings containing seven subunits? (Yan *et al.*, 2018 ▸). It is possible that the oligomer may be wholly or partially generated by the crystal symmetry.
(v) Does the new sequence allow the prediction of secondary-structure elements, and if sequence searches only find structures with low sequence homology, do the sequence-based alignments show any agreement with the secondary-structure predictions? Such analyses are possible using *AMPLE* (Rigden *et al.*, 2018 ▸) or *Rosetta* (DiMaio *et al.*, 2011 ▸).

## 4. The properties of the diffraction images and the crystal lattice   

After growing a crystal and collecting and processing data, there is more information to consider before attempting a molecular-replacement calculation.

### 4.1. What is the quality of the experiment?   

Luckily, there are certain standard properties of diffraction which help to judge this. [The *CCP*4*i*2 data-processing reports (Potterton *et al.*, 2018 ▸) provide a detailed analysis of these issues.]

What is the completeness and resolution of the diffraction data? If there are blocks of unobserved data, this can hamper any molecular-replacement search.

Are the data very anisotropic? If so, it may be easier to solve and refine the structure if the data are truncated.

Could the crystal be twinned? This can make point-group assignment difficult, but molecular-replacement searches can usually be successful with such data.

If the resolution is low, perhaps limited to less than 3 Å, the rebuilding of the model will be more difficult.

### 4.2. Deciding the point group, asymmetric unit contents and possible space group   

It is usually possible to determine the point group of the crystal unambiguously from the diffraction symmetry (if there is no twinning). This allows the volume of the asymmetric unit to be calculated, and hence the Matthews coefficient (Matthews, 1968 ▸), which gives an estimate of the likely number of molecules in that volume, assuming the solvent volume in the crystal. Most crystals contain about 50% solvent, but there are exceptions, for example the crystal structure in PDB entry 5lf5 has 90.3% solvent (Pronker *et al.*, 2016 ▸) while that in PDB entry 2yln has 26.4% solvent (Bulut *et al.*, 2012 ▸). It is of course more difficult to predict the number of copies in the asymmetric unit accurately as the number increases.

An initial **guess** of the likely space group(s) can be made on the basis of the systematic absences.

For example, if there is threefold symmetry in one reciprocal-lattice plane then the point group is *P*3. Possible space groups are then *P*3, *P*3_1_ or *P*3_2_. If the symmetry operators relate atom (*x_j_*, *y_j_*, *z_j_*) to atoms (−*y_j_*, *x_j_* − *y_j_*, *z_j_* + 1/3) and (−*x_j_* + *y_j_*, −*x_j_*, *z_j_* + 2/3) or atom (*x_j_*, *y_j_*, *z_j_*) to atoms (−*y_j_*, *x_j_ −* *y_j_*, *z_j_* + 2/3) and (−*x_j_* + *y_j_*, −*x_j_*, *z_j_* − 1/3) then only the reflections (0, 0, *l*) where *l* equals 3*n* will be observed and the probable space group is equally likely to be either *P*3_1_ or *P*3_2_. These space groups are called enantiomorphs.

### 4.3. Are there noncrystallographic operators relating molecules?   

If there is more than one molecule per asymmetric unit, the diffraction data can be analysed to provide some clues to their relative orientation.

#### 4.3.1. Noncrystallographic translations   

A Patterson map calculated using the observed intensities may show a strong noncrystallographic translation vector at (*x*_nc_, *y*_nc_, *z*_nc_), indicating that some pairs of molecules are oriented in the same way relative to the crystal axes but one is translated relative to the other by (*x*_nc_, *y*_nc_, *z*_nc_). This information can be misleading for space-group determination. For example, if *z*_nc_ is 1/3 then even if the true space group is *P*3, only (0, 0, *l*) reflections with *l* = 3*n* will be observed.

Such noncrystallographic translations introduce severe structure-factor correlations which affect the statistical analyses to detect twinning, and other anomalies (Read *et al.*, 2013 ▸), and if left uncorrected degrade the scoring functions used to judge molecular-replacement solutions (Jamshidiha *et al.*, 2019 ▸).

#### 4.3.2. Is there other noncrystallographic symmetry?   

**Figure 2:**
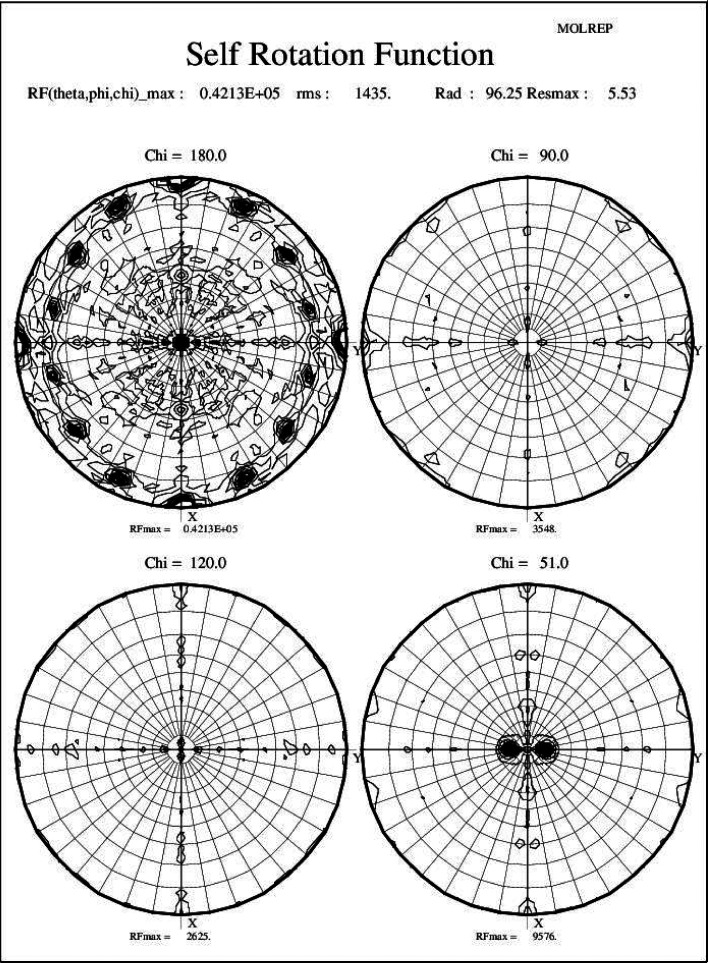
GroEL self rotation. Plots based on the data for PDB entry 5opx. The section at χ = 180° shows seven peaks relating the seven copies of GroEL to their symmetry pairings, and the section at χ = 51° (∼360/7) shows the directions of the two sevenfold axes.

The Patterson vectors generated for each molecule will be related and this feature can be analysed using a self-rotation function. If this is present, the oligomer symmetry may complicate the interpretation of the crystal symmetry (Fig. 2 ▸).

## 5. Is it possible to position a starting model in the crystal lattice? The molecular-replacement search   

Sensible initial checks are the following.

(i) Is this data set the same as a deposited data set? *i.e.* have I collected lysozyme data AGAIN? (Keegan *et al.*, 2018 ▸).
(ii) Is the model in the same space group as, with similar cell dimensions to, the new data?

If so, there is no need to carry out an MR search; it is sufficient to start refinement from the existing model (possibly after reindexing the data, if there are alternative ways to index data in the space group), changing the sequence where necessary, and proceed to rebuilding.

### 5.1. Basics of molecular replacement   

If neither of the above is the case, then it is necessary to use **molecular-replacement techniques** to find possible starting positions for the model and a **scoring system** to rank likely solutions. These procedures are covered in detail in previous CCP4 Study Weekend publications. There is an excellent introduction in Evans & McCoy (2008 ▸).

We need to define a rigid rotation to correctly orientate the model relative to the new crystal axes, and possibly a translation to move the model to a position in the new cell consistent with the crystal symmetry.

Mathematically, this can be written as

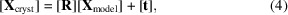

where [**R**] is a rotation matrix and [**t**] is a translation vector, *i.e.*


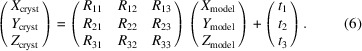




When considering the rotation matrix, it is convenient to consider the coordinates *X*_cryst_ and *X*_model_ as given relative to an orthonormal axial system **X**, **Y**, **Z**. Most molecular-replacement software defines the orthonormal axes to be **X** parallel to **a**, **Z** parallel to **a** × **b** and **Y** in the **ab** plane.

**Rotation matrices** have well defined properties. They can be expressed as a function of **three rotation angles** only. There are various conventions for selecting the rotation angles; the most widely used are Eulerian angles (α, β, γ). Details of the different conventions are described in Evans (2001 ▸).

The **translation vector** positions the rotated molecule in the unit cell relative to certain **symmetry rotation axes**. (In fact, it is easier to think of this vector in terms of fractional shifts along the crystal axes.)

In space group *P*1 there is no rotational symmetry, so the vector [**t**] can take any value because the relative positions of atoms in the crystal remain unchanged.

For polar space groups such as *P*2_*i*_, *P*3_*i*_, *P*4_*i*_ and *P*6_*i*_ it is only necessary to fix two parameters of [**t**], since any position along the polar axis can be chosen without changing the relative positions of atoms in the crystal.

For all other space groups with intersecting symmetry operators it is necessary to fix all three parameters of [**t**].

It is not usually feasible to simply check all values of these parameters and choose the ‘best’ result; even with modern computers the time taken would be astronomical.

The first simplification to speed up the search is to break it into two parts: first to find a range of likely rotation angles and then to restrict the translation search to the orientations defined by these.

## 6. How best to determine these parameters?   

The simplest thought experiment to help to visualize these procedures is to consider them as a matching of Patterson map volumes.

### 6.1. The rotation function   

Hoppe (1957 ▸) compared Patterson maps calculated for known chemical fragments with the observed Patterson maps for larger molecules. He traced these onto transparent paper and matched them by eye to determine the positions of the fragment in the unit cell.

Rossmann & Blow (1962 ▸) independently developed a computer-based method for obtaining likely rotation angles. They found the best fit of the model and crystal Patterson maps over a spherical volume centred at the origin as the model Patterson map was rotated. Since the search was restricted to a spherical volume, the Patterson map could be expressed using spherical harmonics and the calculations were all carried out in reciprocal space. Later, this allowed fast Fourier transforms (FFTs) to be exploited to generate the full range of maps for all rotation angles (Crowther & Blow, 1967 ▸; Navaza, 1994 ▸; Vagin & Teplyakov, 1997 ▸).

The likelihood-based fast rotation function used in *Phaser* weights the observations taking into account crystallographic and noncrystallographic symmetry and the actual unit cell. The calculated Patterson map is appropriately weighted to reflect the model accuracy. Consideration of the likely data distributions and model errors also allows a prediction of whether a solution is likely to be found before starting the search.

The form of the approximation is chosen so that it can be computed using spherical harmonics, which yields weighted Patterson-like coefficients, which are used in an analogous way to Patterson-based methods (McCoy *et al.*, 2007 ▸).

### 6.2. The translation function   

If the crystal lattice exhibits rotational symmetry, the correctly orientated model must also be correctly positioned in the unit cell relative to these symmetry axes.

When the model is moved by some translation then the symmetry-related copies will also move, and a second Patterson search can be used to suggest a likely translation. The pattern of intermolecular vectors between the symmetry copies can be predicted, but the centre of the constellation will change as the reference structure is moved relative to the crystal origin. The required translation can be found by translating the intermolecular vectors over the observed Patterson map and computing another Patterson product function. When the correct translation is chosen, this should be large because the vector sets will coincide.

The maximum-likelihood-based fast translation search uses similar approximations to those for the fast rotation search. Likely solutions are then rescored using a likelihood-weighted correlation between calculated and observed intensities.

## 7. Scoring systems for the molecular-replacement search   

### 7.1. How best to reject wrong ‘solutions’?  

(i) The simplest ‘scoring system’ is to reject these ‘solutions’ where, after positioning the model, there are multiple clashes between the symmetry-related copies.
(ii) If the solution is incorrect, the calculated structure-factor amplitudes will not show any agreement with the measured ones. This means that wrong solutions cannot be refined by standard procedures. If the model is poor, even a correct solution will generate almost random starting *R* factors (*i.e.* ∼55%), but if initial refinement cycles cannot reduce these *R* factors to below 50% then the solution is probably wrong.

### 7.2. How best to recognize correct solutions?   

#### 7.2.1. Can the new structure be refined and rebuilt?   

This is obviously the most important criterion of success. Electron-density maps generated using calculated phases from a partial model should show where corrections need to be made. If the initial *R* factors derived from the model decrease significantly in the initial cycles of refinement then the model is likely to be sufficiently accurate to allow rebuilding, either automatically or by hand.

#### 7.2.2. Log-likelihood gain on intensities (LLGI)   

Likelihood is the probability that the experimental data measurements could be predicted given a particular model. It provides a tool to compare how well different models agree with the data. (In the case of molecular replacement, the model to be assessed is the atomic coordinates after selected rotation and/or translation operators have been applied.) LLGI is the difference between the likelihood of the current model predicting the observed intensities and the likelihood based on a random distribution derived from a Wilson distribution of intensities. It scores how much better the observations can be predicted using your model rather than from a random distribution of the same atoms (Oeffner *et al.*, 2018 ▸; Read & McCoy, 2016 ▸).

This is a much more sensitive measure of success than the earlier Patterson-based correlation estimates. It takes into account the completeness of the search model, the likely root-mean-square difference (r.m.s.d.) between the model coordinates and those of the new molecule, and the accuracy of the measured intensities, whilst accounting for the effects of certain common pathologies, such as anisotropy, noncrystallographic translations and twinning.

The absolute value of the LLGI for a given solution is a measure of how probable it is that the solution is correct. It is also possible to predict the expected LLGI that will be achieved from a given model (eLLG). Assuming a certain r.m.s.d. between the model and the target structure (which can be estimated from the sequence identity), it is possible to rank models and tailor search strategies to the difficulty of the molecular-replacement problem. Of course, there are still uncertainties; the model error can usually only be estimated from the sequence match, and the true error may vary considerably from this estimate.

#### 7.2.3. The *Z*-score   

The *Z*-score, which shows how many standard deviations of LLGI a particular solution is above the mean LLGI, provides a quick measure of success. A score of 8 or above usually indicates a correct solution.

#### 7.2.4. Patterson overlap   

This is still used in most software packages to select a range of likely rotation-function solutions to score. Initially, the overlap was measured by a simple product function; later, more sophisticated weighting schemes were incorporated in the *X-PLOR* package.

## 8. Examples   

To illustrate these points, I will consider the following structures. Full details are given in Table 1 ▸. The following examples are chosen to illustrate some of the issues raised in the above text.

### 8.1. Consider the known chemistry   

The calcium-free S100 protein, PDB entry 2wce, is part of a large family of calcium-binding proteins (Moroz *et al.*, 2009 ▸). It is well known that when calcium binds these proteins undergo a large domain movement. However, automated searches for suitable models based on sequence alone cannot use this information.

The experimental data extend to 1.8 Å resolution and the models with PDB codes 1k96 and 1k9p both have the same sequence, with 38% sequence identity to PDB entry 2wce (Otterbein *et al.*, 2002 ▸). PDB entry 1k96 has calcium bound, whilst PDB entry 1k9p is calcium-free, and the r.m.s.d. between their C^α^ positions is 1.95 Å. PDB entry 2wce is easily solved using PDB entry 1k9p as a model, but the search fails when PDB entry 1k96 is used because of the conformational change.

### 8.2. A straightforward case   

The isomerase, PDB entry 1vky, has X-ray data to 2.2 Å resolution, and there is a satisfactory model, PDB entry 1yy3, with 38% sequence identity (Mathews *et al.*, 2005 ▸; Grimm *et al.*, 2006 ▸). Although the reported LLG is low, the solution is straightforward; the initial *R* values of 55% fall to 50% and 52% after refinement; the initial phase error is 62° and the *Buccaneer* pipeline (Cowtan *et al.*, 2011 ▸) builds much of the structure automatically.

The sequence alignment between PDB entries 1yy3 and 1vky shows that model residues 130–279 have a higher sequence identity (53%) than for the whole model. In fact, searching with this truncated model gives a better result; the LLG scores are higher and the initial phase error is lower. Again, the *Buccaneer* pipeline builds most of the structure from this truncated model.

### 8.3. Oligomers   

All haemoglobins form a dimer of dimers, each containing related chains A and B, each of which carries a haem molecule. PDB entry 4hhb is the model of human deoxyhaemoglobin with the complete tetramer in the crystal asymmetric unit (Fermi *et al.*, 1984 ▸). When oxygen binds to the haem there is a 15° rotation between the dimer pairs. The A and B chains have a sequence identity of 45%. The model is taken from PDB entry 1hho: the structure of human oxyhaemoglobin with an identical sequence. In this structure the asymmetric unit contains one AB dimer, and the tetramer is generated by a crystallographic twofold rotation.

When the high-resolution (1.7 Å) PDB entry 4hhb data are searched using the AB dimer from PDB entry 1hho (Shaanan, 1983 ▸), the solution is spectacularly clear; the final LLG is 3042 and the initial structure refines to an *R* and *R*_free_ of 28% and 32%, respectively.

Even when the search is carried out using the A chain alone the solution is very obvious, with the LLG steadily increasing as each chain is positioned. Subsequent refinement and automated rebuilding corrects the A-chain sequence to the required B-chain sequence.

Surprisingly, a solution can be found starting from a search model of a 12-residue idealized helix representing about 3% of the molecule. This shows the power of *Phaser* discrimination. 11 helices can be placed, which is sufficient to kick-start rebuilding.

It is worth noting that the rebuilding procedure progresses much more smoothly when the Fe atoms and the haem group are positioned into the initial maps and then held fixed. In this case, the first map from the molecular-replacement search was sufficiently clear to allow this to be performed.

### 8.4. High-resolution solutions   

The final and simplest example is PDB entry 6cum (Abendroth *et al.*, 2018 ▸). This is an engineered 52-residue protein which was predicted to be mostly helical. The resolution of the deposited data is 1.60 Å, although the diffraction could probably have been extended. *Phaser* positioned two 12-residue helices, only one correctly. Density modification using *ACORN* (Jia-xing *et al.*, 2005 ▸) reduced the phase error from 70° to 36°, and not surprisingly the rebuilding was extremely straightforward.

These examples illustrate a few general considerations.

Firstly, it **really** helps to have higher resolution experimental data.

Secondly, the scoring system based on LLGI is very sensitive to a realistic estimate of the r.m.s.d. between model and molecule C^α^ atoms. This is obviously very accurate for models of ideal α-helices, but is not necessarily so for larger proteins with domain movements. The careful inspection of a range of models could help to eradicate flexible regions. Better results may be obtained from a smaller but more accurate model.

Thirdly, if the molecule contains heavy atoms or bulky ligands it assists rebuilding if these are positioned and fixed as early as possible.

## 9. A brief historical overview   

The rotation function, the tool used to determine the orientation of two related molecules by searching for matching features in Patterson maps, was first suggested by Hoppe (1957 ▸). His *Faltmolekul Methode* found the skeleton of small molecules in a related crystal, and Huber (1965 ▸) used this technique to solve the structure of an insect hormone, ecdysone, by searching with a model constructed from a steroid moiety.

However, the rotation and translation functions as proposed by Rossmann & Blow (1962 ▸), or the faster versions described by Crowther (Crowther & Blow, 1967 ▸; Crowther, 1972 ▸), were the usual tools used for proteins. The original molecular-replacement program developed by Michael Rossmann and David Blow used a simple Patterson overlap function, measured by a product function of the corresponding positions within a sphere of pre-selected volume centred at the origin of the map and edited to exclude the Patterson origin peak.

The translation function overlapped Patterson volumes away from the origin to try to find relative shifts from one molecule to another in the unit cell.

The first use of the technique for proteins was just to identify noncrystallographic symmetry operators relating the orientations of different molecules in a crystal asymmetric unit (Rossmann & Blow, 1962 ▸; Dodson *et al.*, 1966 ▸). In the first studies, the method was applied to crystals where it was known that the asymmetric unit of the crystal contained two or more copies of the molecule under investigation. In this case, the overlap of the observed Patterson on itself after some rotation should be maximum when that rotation matches the vector patterns generated by the different copies of the molecule. In fact, when we reported to Dorothy Hodgkin that we had ‘proved’ that 2Zn insulin crystallized with 32 symmetry, but the twofold axis in 4Zn insulin did not intersect the crystallographic threefold axis, she said ‘But surely you can **see** that in the Patterson maps’, and indeed she was right, but the program proved to be useful in more complex cases.

When a model was available, the product function was calculated between the observed Patterson map and the calculated Patterson map for that model. In general, the higher the crystal symmetry, and the more molecules to search for, the harder it was to find a clear solution for the rotation function. However, for the translation function, the more symmetry operators the clearer the solution could be.

By the 1970s, we were able to position the coordinates of a related structure in a new unit cell using the methodology developed by Crowther and Blow and encapsulated in the program *ALMN* to find the rotation angles, and a slow *R*-factor search of the correctly orientated molecule moved over a relatively coarse grid covering the crystal asymmetric unit (Crowther, 1972 ▸; Nixon & North, 1976 ▸). This was obviously quicker to calculate when the crystal and oligomer symmetry allowed you to reduce the search volume to a single 2D section.

By the 1980s more automated pipelines had become available, although these were often not reported in the literature until much later. The most widely used were probably *MERLOT*, developed by Paula Fitzgerald (Fitzgerald, 1988 ▸), *MOLREP*, developed by Alexei Vagin (Vagin & Teplyakov, 1997 ▸), and *AMoRe*, developed by Jorge Navaza (Navaza, 1994 ▸). In these pipelines, each step of the procedure was programmed separately, but the output of each fed seamlessly into the next stage. Jorge Navaza found that the correlation coefficient between the observed amplitudes for the crystal and the calculated amplitudes from even a single copy of a correctly orientated model was an effective discriminator, even though those amplitudes were generated without accounting for the symmetry copies. *AMoRe* also contained a very effective *FITFUN* module which checked for model overlaps and refined the rotation and translation solutions by maximizing the correlation coefficients between observed and calculated amplitudes.

Axel Brünger exploited a more sophisticated Patterson correlation coefficient in *X-PLOR* to rank rotation-function solutions. This used normalized structure factors and extended parametrization of the model (Brünger, 1990 ▸).

It is interesting to follow the developments in this technique as charted in the *Proceedings of the CCP4 Study Weekend*. The first meeting devoted to MR was held in 1985,^1^ with 83 participants; by this time it was established as a useful tool for structure solution. There were presentations from David Blow, Phil Evans, Ian Tickle and myself, showing off our hard-won basic mathematical knowledge, defining axial systems, parameters for rotation matrices, spherical harmonics, fast Fourier implementations, the interaction of noncrystallographic and crystal symmetry, and so on. (Nowadays these issues are taken for granted.) There was discussion of the problems introduced by incomplete data, gross measurement errors and high temperature factors, but without any systematic agreed solution. Lots of case studies were presented, mostly beginning by thanking the friend who had supplied the coordinates of a related molecule. At that time, the PDB archive was generally too limited to provide a suitable model. The programs used were *ALMN* for rotation searches, extended from Tony Crowther’s work, and to pinpoint the translation vector, *TRANS*, which performed a Patterson search, or *RSEARCH*, which used FFTs to calculate structure factors over a grid covering the crystal asymmetric unit. Various contributors, including me, discussed possible scoring functions; for example, reject clashing solutions, or only believe a solution when the model phases allow you to (i) position heavy atoms and (ii) rebuild and refine the new crystal form.

By the time of the next Study Weekend on MR in 1992, there were several bioinformatic discussions describing ways to use the rapidly expanding PDB archive. There were descriptions of new software available for MR pipelines [*MERLOT*, *X-PLOR* (Brünger, 1990 ▸), *AMoRe* and *MOLREP*]. Several papers discussed how to proceed from a solution; there were new methods for averaging electron-density maps to improve phases, new maximum-likelihood-based refinement programs were becoming available, and graphics facilities were rapidly improving.

In 2001 (Naismith *et al.*, 2001 ▸), Randy Read described weighting schemes based on multivariate statistics to generate more realistic models and maximum-likelihood scoring functions for rotation and translation searches. There were contributions describing the use of novel ‘models’; for example, EM images, NMR models and blocks of electron density. The existing software was being improved and extended, and there were discussions of new features in *AMoRe*, *CNS*, *Queen of Spades* and *GRLF* (the ‘locked’ rotation function).

The 2008 meeting (Murshudov *et al.*, 2008 ▸) provided a most valuable set of reference papers. There was a comprehensive and clear introduction to the technique by Evans & McCoy (2008 ▸), and the first discussions of pipelines such as *MrBUMP* and *BALBES* which included a bioinformatic search for a model.

The 2013 meeting (Ballard *et al.*, 2013 ▸) included an excellent paper by Oeffner, Bunkóczi, McCoy and Read titled *Improving estimates of coordinate error for molecular replacement* (Oeffner *et al.*, 2013 ▸). There were the first discussions of generating models from sequence information alone, and examples of successful MR searches using models generated by *Rosetta* and other related morphing/model-construction tools. The first reports of the solution of structures from relatively tiny fragments were presented.

By 2020, 86% of the structures deposited in the wwPDB were being solved by MR, which has become such a powerful tool because of several interlocking developments. The wwPDB now provides a fantastic resource covering many, many structural families, and the sequence-searching and structure-prediction tools are superb. Powerful synchrotron resources mean that the quality of the measured data is enhanced, and thus correcting the initial model is more straightforward. At the same time the computing power routinely available means that multiple ‘solutions’ can be assessed, a small fraction of which may be usable as starting points for structure determination.

## 10. Conclusion   

Molecular-replacement techniques will continue to underpin the majority of crystal structure solutions, and automated pipelines will mean that there will be less interest in these basic equations, and more study of improved bioinformatics tools for model selection and of techniques for structure completion. As the underlying databases are expanded, and the experimental data quality is improved, these pipelines will also provide better results. The interplay of crystallography and electron microcroscopy will provide new challenges.

## Figures and Tables

**Figure 3:**
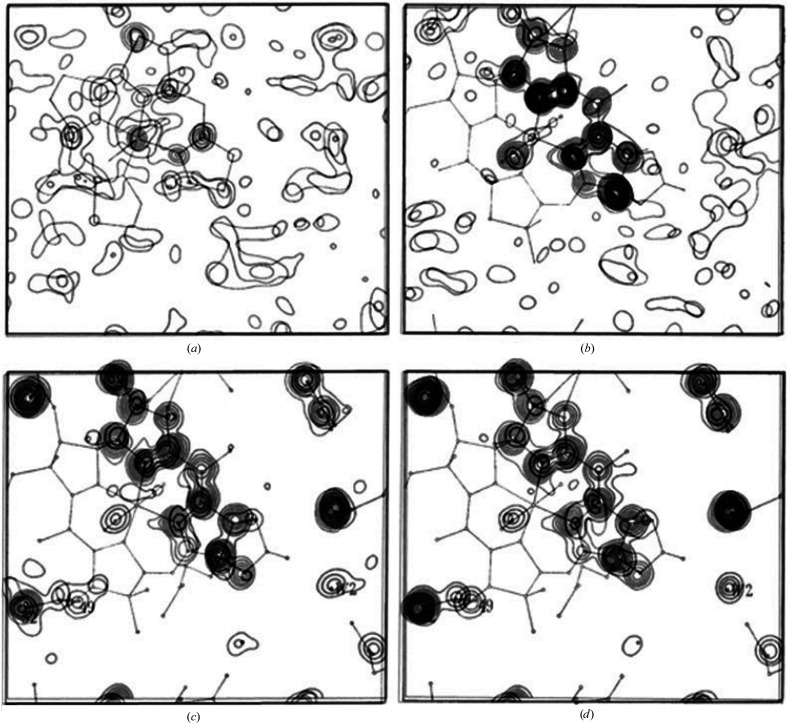
Illustration of bootstrapping from a partial solution: progressive vitamin B_12_ Fourier maps showing density for the corrin ring. (*a*) Co only. (*b*) Co + eight atoms. (*c*) Co + 17 atoms. (*d*) Complete.

**Table 1:** Example reports The column headings are as follows. Mol, PDB identifier for the test structure. Nres, number of residues in the test-structure molecule. Nmol, number of molecules in the crystal asymmetric unit. SG, space group. Model, the identifier of the structure used as a search model for MR. Nres, number of residues of the model used in the MR search. Seq_ID, sequence ID between model and test structure. RmsCA true/est, the r.m.s.d. between the aligned C^α^ atoms of the model and the test structure; true gives the actual value and est the estimated r.m.s.d. based on the sequence identity. eLLG, the estimated LLG based on sequence identity. LLGI, the LLGI values as each copy of the search moiety is placed and, in bold, the final LLGI after model refinement against all observations. *R*, *R*_free_ init/ref, the initial *R* and *R*_free_ for the solution (init) and those obtained after ten cycles of refinement (ref). Dphi_0, the phase error between the correct value and the phases generated from the best solution; Dphi_DM, the phase error after density modification, which was performed with *Parrot*, except for PDB entry 6cum, which used *ACORN*. Rebuilt?, Yes if the test structure could be rebuilt.

Mol	Res	Nres	Nmol	SG	Model	Nres	Seq_ID	RmsCA true/est (Å)	eLLG	LLGI	*R*, *R*_free_ init/ref	Dphi_0/Dphi_DM (°)	Rebuilt?
1vky	2.2	288	2	*I*222	1yy3	253	0.38	1.8/1.1	216	37/90	55/50	62/57	Yes
										**123**	55/52		
1vky	2.2	288	2	*I*222	1yy3, residues 130–279	149	0.53	1.1/0.9†	126	81/309	54/48	53/47	Yes
										**319**	53/51		
2wce	1.8	93	2	*H*3	1k9p	91	0.38	1.2/0.9	79.8	35/82	52/52	76/66	Yes
										**85**	46/49		
2wce	1.8	93	2	*H*3	1k96	91	0.38	1.9/0.9†	79.8	21/52	52/51	89/89	No
										**55**	50/51		
4hhb	1.7	143	2	*P*2_1_	1hho, chains A and B	478	1.0	0.5/0.8	1978	912/3016	30/34	53/35	Yes
		146	2							**3042**	28/32		
4hhb	1.7	143	4	*P*2_1_	1hho, chain A	143	1.0/0.45	0.6/1.5	597.0	179/567/762/977	45/46	57/50	Yes‡
		146								**2184**	37/40		
4hhb	1.7	289	2	*P*2_1_	Helix	12	—	0.3	1.5	63/…	50/51	66/65	Yes
										**780** §	48/50		
6cum	1.6	51	1	*P*3_1_2_1_	Helix	12	—	0.3	37.2	89/105	55/53	70/36¶	Yes
										**106**	56/57		

† For PDB entry 2wce the RmsCA estimate based on sequence identity was too low when using PDB entry 1k96 as a model, and for PDB entry 1vky it was too low when using the whole of PDB entry 1yy3 as a model. It was clear that PDB entry 1k96 would not be a good model, since it bound calcium, whilst PDB entry 2wce did not. The PDB entry 1vky MR search results were better with the partial model of PDB entry 1yy3, using only residues 130–279, than for the search using all of PDB entry 1yy3. This could have been predicted by more careful inspection of the sequence alignment.

‡ The rebuilding benefited from a preliminary inclusion of the HEM entities in the initial model.

§ The power of *Phaser *to position 12-residue α-helices in PDB entry 4hhb is impressive. The LLG is only given for the first placement (28) and the final (eleventh) placement (786).

¶ The impressive phase improvement for PDB entry 6cum from 70° to 36° was achieved by applying the *ACORN* density-modification procedure.

## Footnotes

1 The contents of the 1985 and 1992 *Proceedings of the CCP4 Study Weekend* are available from the website https://epubs.stfc.ac.uk by searching for DL/SCI/R33 for the 1992 meeting or DL/SCI/R23 for the 1985 meeting.

## Appendix A The wonders of crystallography: why ‘bootstrapping’ is possible

Before discussing the techniques and scoring systems used for molecular replacement, it is useful to remind ourselves of the fundamental crystallographic equations. (In the following sections, vectors are represented in bold font and magnitudes in plain font.)

### A1. Properties of a crystal

A crystal is an ordered array of atoms repeated regularly by translation in three directions. These translations define the crystal lattice. The three lattice vectors and the angles between them are conventionally labelled (**a**, **b**, **c**, α, β, γ).

Crystals are described in terms of their unit-cell content, which is the smallest part of a crystal that, if repeated regularly by translation in three dimensions, creates the whole crystal.

The position of each atom within the unit cell can be given as (*x*_*j*_, *y*_*j*_, z_*j*_), where *x_j_*, *y_j_*, *z_j_* are fractional coordinates of the lattice vectors relative to some chosen origin. The vector from the cell origin to the atom position is **x**_*j*_ = *x_j_* **a** + *y_j_* **b** + *z_j_* **c**.

It is possible to maintain a periodic distribution in three dimensions whilst incorporating certain symmetry relationships between several molecules within each unit cell. Molecules can only be related by *n*-fold rotational symmetry, where *n* is 2, 3, 4 or 6, and by screw translations of *m*/*n* along the axes **a**, **b**, **c**, where *m* = 1, 2, …, *n* − 1 (Schönflies, 1891 ▸). The crystal origin is conventionally chosen relative to these symmetry axes.

#### A1.1. Diffraction

The crystal lattice acts as a diffraction grating and thus, when an X-ray beam is shone onto the crystal, the reflected beam is enhanced in certain directions. This diffraction pattern can be conveniently indexed as ‘reflections’ (*h*, *k*, *l*) relative to ‘reciprocal-lattice axes’ defined as (**a***, **b***, **c*** , α*, β*, γ*) which satisfy the conditions **a** · **a*** = **b** · **b*** = **c** · **c*** = 1 and **a** · **b*** = **a** · **c*** = **b** · **a*** = **b** · **c*** = **c** · **a*** = **c** · **b*** = 0.

The coefficients *h*, *k* and *l* can only take integer values, and the intensity *I*(*hkl*) is observed at the reciprocal-lattice vector **h** = *h* **a*** + *k* **b*** + *l* **c***.

The symmetry within the crystal is matched by symmetry within the reciprocal lattice.

### A2. Structure-factor equation

If all of the *N* atomic positions in the unit cell are known, then the magnitude of the calculated structure factor **F**_calc_(*hkl*) will match that of the diffracted **F**_obs_(*hkl*).

**F**_calc_(*hkl*) is the complex sum of the scattering from all *N* atoms in the cell. If *S* is a function of the resolution of this reflection and the atom has scattering power *f*_*j*_(*S*), temperature factor *T_j_* (*S*) and fractional positions (*x_j_*, *y_j_*, *z_j_*), it can be written as

where *g_j_* (*S*) = *f_j_* (*S*)*T_j_* (*S*).

### A3. Effects of symmetry

If the crystal lattice has internal symmetry, *i.e.* is not in space group *P*1, then some sets of these atom positions are related by the symmetry operators; for example, if the space group of the crystal is *P*2_1_ with the screw axis along the *y* axis (as in the conventional setting), then for the *N*/2 atoms at positions (*x_j_*, *y_j_*, *z_j_*) there are *N*/2 related atoms at positions (−*x_j_*, *y_j_* + 1/2, −*z_j_*).

### A4. Origin shifts

If all atoms (*x_j_*, *y_j_*, *z_j_*) in the cell are moved by a fixed amount (*x*_0_, *y*_0_, *z*_0_) then

*i.e.* the magnitude of **F**_calc_(*hkl*) has not changed but the phase has changed by φ(*hkl*)(0).

This can lead to confusion about ‘choosing an origin for the model coordinates’. If there is no crystal symmetry to consider then the choice of origin is arbitrary and (*x*_0_, *y*_0_, *z*_0_) can take any values, but if there is internal crystal symmetry then it is customary to choose an origin on a symmetry axis; for example, for space group *P*2_1_ anywhere along the twofold axis parallel to the crystal **b** axis, or for space group *P*222 at the intersection of the three twofold axes. However, there are often several choices, for example in *P*222 three twofold axes intersect at (0, 0, 0) or (1/2, 0, 0) or (0, 1/2, 0) *etc.*, so the conditions for a ‘solution’ are satisfied equally by (*x_j_*, *y_j_*, *z_j_*) or (*x_j_* + 1/2, *y_j_*, *z_j_*) or (*x_j_*, *y_j_* + 1/2, *z_j_*) *etc.* (The CCP4 documentation provides a useful table of these: http://legacy.ccp4.ac.uk/html/alternate_origins.html.)

When comparing MR solutions obtained from different search procedures, it is sensible to relate them to the same origin, and there are a variety of programs available which do this, for example *CPHASEMATCH*, *phenix.famos*, *CSYMMATCH* *etc.*

### A5. Electron-density maps

The equation for the electron density is used to compute its value at **discrete regular divisions** (grid points) of the unit cell. If the phases are accurate, there will be a peak in the density when the **map coordinate** (*x*, *y*, *z*) is close to the **model coordinate** (*x_j_*, *y_j_*, *z_j_*).

summed over all *h*, *k* and *l*.

An error-free observed *F*_obs_(*hkl*) and φ(*hkl*) calculated from a complete, error-free model will generate a perfect observed electron-density map showing the position of every atom in the molecule.

However, neither the observations nor the model are likely to be complete or perfect.

Consider the map

where φ_part_(*hkl*) is calculated from an imperfect model which has missing and/or misplaced atoms.

Since *F*_obs_(*hkl*) contains information about the total model, this map will show peaks for these missing atoms at something less than half their expected height.

#### A5.1. Maximum-likelihood-weighted difference electron-density maps

Consider

where *k* is the scale factor required to adjust the observed amplitudes measured on an arbitrary scale to a value which best matches the calculated amplitudes. (It is not always trivial to find the best value for *k*.) *M*(*hkl*) is a weight assigned to each *F*_obs_, and *D*(*S*) is a σ_A_ weight reflecting the fit of the model to the observations at resolution *S*.

#### A5.2. Patterson maps

Using the equation for **F**(*hkl*) we can show

Thus, calculating a map replacing **F**(*hkl*) with *F*(*hkl*)*F**(*hkl*) and with **all phases zero** gives a map with peaks at all positions (**x**_*i*_ − **x**_*j*_), *i.e.* at the vector difference between any two atoms **x**_*j*_ and **x**_*i*_. Patterson interpretations can kick-start many phasing procedures (Patterson, 1934 ▸).

In the vitamin B_12_ study illustrated in Fig. 3 ▸, the first phase information was generated from a single heavy Co atom (16% of the total scattering), which was positioned from the Patterson map. The peak height for the Co–Co vector is by far the strongest in this map (Hodgkin *et al.*, 1955 ▸).

The interatomic vectors will include vectors between atoms in the same molecule (intramolecular vectors) and vectors between atoms in one molecule and atoms in its symmetry or lattice-shifted repeat (intermolecular vectors). In general, vectors within the same molecule are shorter and are therefore likely to be clustered around the Patterson map origin.
